# Influence of Quaternary environmental changes on mole populations inferred from mitochondrial sequences and evolutionary rate estimation

**DOI:** 10.1186/s40851-021-00169-9

**Published:** 2021-02-15

**Authors:** Azusa Nakamoto, Masashi Harada, Reiko Mitsuhashi, Kimiyuki Tsuchiya, Alexey P. Kryukov, Akio Shinohara, Hitoshi Suzuki

**Affiliations:** 1 Graduate School of Environmental Science, Hokkaido University, North 10, West 5, Sapporo, 060-0810 Japan; 2 Laboratory Animal Center, Osaka City University Graduate School of Medical School, Osaka, 545-8585 Japan; 3 Oyo-seibutsu Co. Ltd., 4-12-3, Minami-Aoyama, Minato-ku, Tokyo, 107-0062 Japan; 4 Federal Scientific Center of the East Asia Terrestrial Biodiversity, Far Eastern Branch of the Russian Academy of Sciences, Vladivostok, 690022 Russia; 5 Frontier Science Research Center, University of Miyazaki, Kihara 5200, Miyazaki, 889-1692 Japan

**Keywords:** *Mogera*, Phylogeography, Japanese archipelago, Cytochrome *b* gene, Time-dependent evolutionary rate

## Abstract

Quaternary environmental changes fundamentally influenced the genetic diversity of temperate-zone terrestrial animals, including those in the Japanese Archipelago. The genetic diversity of present-day populations is taxon- and region-specific, but its determinants are poorly understood. Here, we analyzed cytochrome *b* gene (*Cytb*) sequences (1140 bp) of mitochondrial DNA (mtDNA) to elucidate the factors determining the genetic variation in three species of large moles: *Mogera imaizumii* and *Mogera wogura*, which occur in central and southern mainland Japan (Honshu, Shikoku, and Kyushu), and *Mogera robusta*, which occurs on the nearby Asian continent*.* Network construction with the *Cytb* sequences revealed 10 star-shaped clusters with apparent geographic affinity. Mismatch distribution analysis showed that modes of pairwise nucleotide differences (τ values) were grouped into five classes in terms of the level, implying the occurrence of five stages for rapid expansion. It is conceivable that severe cold periods and subsequent warm periods during the late Quaternary were responsible for the population expansion events. The first and third oldest events included island-derived haplotypes, indicative of the involvement of land bridge formation between remote islands, hence suggesting an association of the ends of the penultimate (PGM, ca. 130,000 years ago) and last (LGM, ca. 15,000 years ago) glacial maxima, respectively. Since the third event was followed by the fourth, it is plausible that the termination of the Younger Dryas and subsequent abrupt warming ca. 11,500 years ago facilitated the fourth expansion event. The second event most likely corresponded to early marine isotope stage (MIS) 3 (ca. 53,000 years ago) when the glaciation and subsequent warming period were predicted to have influenced biodiversity. Utilization of the critical times of 130,000, 53,000, 15,000, and 11,500 years ago as calibration points yielded evolutionary rates of 0.03, 0.045, 0.10 and 0.10 substitutions/site/million years, respectively, showing a time-dependent manner whose pattern was similar to that seen in small rodents reported in our previous studies. The age of the fifth expansion event was calculated to be 5800 years ago with a rate of 0.10 substitutions/site/million years ago during the mid-Holocene, suggestive of the influence of humans or other unspecified reasons, such as the Jomon marine transgression.

**Supplementary Information:**

The online version contains supplementary material available at 10.1186/s40851-021-00169-9.

## Background

Environmental fluctuations during the Quaternary Period, particularly the 100,000-year cycles of glacial and interglacial intervals, shaped the genetic structure of terrestrial animal populations [1, 2]. Genetic diversity has been substantially affected by local environmental conditions, taxon-specific ecological features, and competition between congeneric species occurring in northern and southern ranges [3]. However, as environmental changes are complex and some are recurring, it is difficult to grasp evolutionary history across space and time. The Japanese Archipelago extends northeast to southwest along the coast of Asia and over a wide range of climatic zones, making it an ideal location to study the evolutionary dynamics of late Quaternary environmental fluctuations. The central domain of the Japanese Archipelago includes four main islands: from north to south, Hokkaido, Honshu, Shikoku, and Kyushu. Many adjacent small islands harbor genetically distinct fossorial and terrestrial mammal species, including dormice [4], moles [5], and shrew moles [6]. During the 100,000-year glacial cycle, these peripheral islands were close to the main island or connected by land bridges, resulting in both migration and isolation [7]. This phenomenon provides a useful metric to calibrate and assess divergence times in phylogenetic analyses conducted for rodent species [3, 7]. It is thus necessary to study other taxa for comparison with rodents.

The impact of prehistoric environmental fluctuations on wood mice and voles in Japan has been elucidated [3, 7, 8]. These studies suggested that three prominent periods affected population dynamics: the last glacial maximum (LGM; 20,000 years ago), marine isotope stages (MIS) 3/4 (53,000 years ago), and the penultimate glacial maximum (PGM; 140,000 years ago) [3, 7, 8]. The large Japanese wood mice (*Apodemus speciosus*) that inhabited the northernmost island, Hokkaido, was heavily influenced by the last glacial period, including the LGM [8]. In contrast, populations on Honshu, Shikoku, and Kyushu were influenced by the PGM but not the LGM [8], probably because the PGM was more forceful than the LGM [9]. Some researchers have hypothesized that forest-dwelling species were more heavily affected by the PGM and LGM, while grassland species were more affected by the transition from MIS 4 to MIS 3 [3, 7, 8, 10]. This difference provides clues to assess the evolutionary rates of time dependence, especially for the early stages of divergence [11–13]. Biogeographic calibration points have been obtained through two independent methods: 1) lineage divergences between remote peripheral islands separated by the deep sea in 100,000-year intervals and 2) population bottlenecks and expansions associated with fluctuating cooling and warming periods during the last 150,000 years [3, 7, 8].

The Quaternary climatic fluctuation influenced several pairs of related species in Japan, including moles *Mogera imaizumii* vs. *M. wogura* [5], voles *Myodes andersoni* vs. *M. smithii* [3, 14], elephants *Palaeoloxodon naumanni* vs. *Mammuthus primigenius* [15], and bears *Ursus thibetanus* vs. *Ursus arctos* [16]. The northern *Myodes* species, *M. andersoni*, expanded its territory to the southern part of Honshu during colder periods and left an isolated population on the Kii Peninsula [14]. During warmer periods, the southern species expanded their distribution northward [3, 14]. We hypothesized that southern species were more susceptible to glacial periods than northern species and that rapid expansion events were more pronounced in southern species. In addition, it is important to better understand the impact of competitive congeneric species in shaping genetic diversity.

**Fig. 1:**
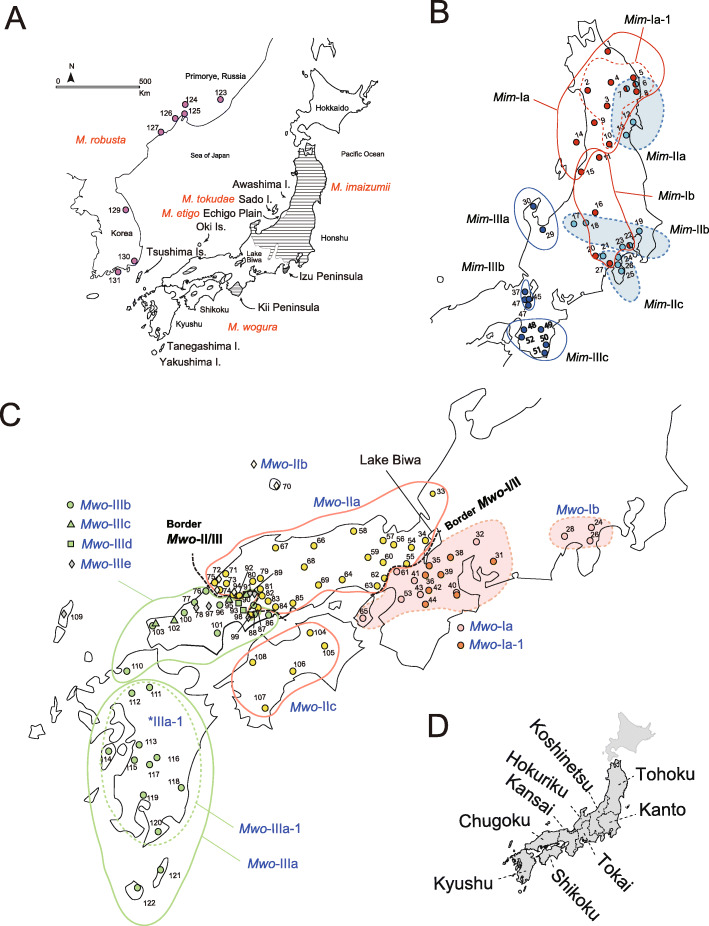
Geographic map of the Japanese Archipelago and nearby Asian continent, showing the collection localities. **a** Distributions of *Mogera imaizumii*, *M. wogura*, *M. robusta*, and collection localities of *M. robusta* (purple) are shown. **b** Collection localities are indicated for intraspecies phylogroups based on cytochrome *b* (*Cytb*) sequences in *M. imaizumii* (*Mim*-I, red; *Mim*-II, light blue; *Mim*-III, blue). **c** Collection localities are indicated for intraspecies phylogroups based on *Cytb* sequences in *M. wogura* (*Mwo*-I, pink; *Mwo*-II, yellow; *Mwo*-III, green). **d** The administrative units of Japan (Tohoku, Kanto, Hokuriku, Koshinetsu, Tokai, Kansai, Chugoku, Shikoku, and Kyushu) are used to describe regionally cohesive sampling localities and phylogroups

Moles are small subterranean mammals that consume earthworms as a major food source and are found in a variety of habitats, including broadleaf forests and paddy fields. East Asian moles (genus *Mogera*) widely inhabit continental East Asia and Japan, Taiwan, and Vietnam [17–20]. There are four large moles on Honshu, Shikoku, Kyushu, and their peripheral islands (e.g., Tsushima Island, Fig. 1a) [20]. The Sado (*Mogera tokudae*) and Echigo (*M. etigo*) moles are confined to Sado Island and a small area of the Echigo Plain, respectively, in northern Honshu [20]. The lesser Japanese mole (*Mogera imaizumii*) is based in northern Honshu, with enclaves in western Honshu and Shikoku. The large Japanese mole (*Mogera wogura*) is found in the Korean Peninsula, East China, and the Maritime region of the Russian Far East (Primorye). Continental populations are now recognized as a distinct species, *M. robusta* [21, 22].

Mitochondrial DNA (mtDNA) variations have been examined in *M. imaizumii* and *M. wogura* [5, 20, 23]. *Mogera imaizumii* contains three major phylogroups with geographic subdivisions in the coastal areas of the Sea of Japan (*Mim*-I), the Pacific Ocean (*Mim*-II), and the central Honshu districts of Hokuriku and Kansai (*Mim*-III). *Mogera wogura* is divided into four groups: those distributed in the Kansai and Tokai Districts (Kansai-Tokai group; *Mwo*-I), in the Chugoku and Shikoku Districts (Chugoku-Shikoku group; *Mwo*-II), in Kyushu and the westernmost tip of Honshu (Kyusyu group; *Mwo*-III), and in China/Korea/Primorye (continent group; *Mwo*-IV; *M. robusta*). However, the geographic borders between the mtDNA lineages are unclear due to limited data [5], and the factors shaping the geographic demarcations are not yet understood. In addition, a comparison with the continental population can provide insight regarding the consequences of environmental fluctuations on allopatric populations.

Here, we examined the population genetic structures of two Japanese mole species, *M. imaizumii* and *M. wogura*, to clarify the geography of mtDNA phylogroups*.* In addition, in *M. imaizumii*, *M. wogura*, and *M. robusta*, we addressed the prominent events of rapid population dynamics with mtDNA sequences, which would be associated with late Quaternary environmental fluctuations. We assigned these rapid expansion events to specific geological times and used them as calibration points to infer the evolutionary rate of mtDNA in comparison with previously obtained data on rodent evolutionary rates in a time-dependent manner [3, 7, 8].

## Materials & methods

### Biological materials

*Mogera imaizumii* specimens (*n* = 77) used for molecular phylogenetic analyses (Supplementary Table S1, Fig. 1) consisted of 40 newly collected individuals and 37 obtained from the GenBank/EMBLE/DDBJ nucleotide database [5, 20, 23, 24]. *Mogera wogura* specimens (*n* = 131; Supplementary Table S1) consisted of 101 newly collected individuals and 30 from the database [5, 20, 24, 25]. *M. robusta* (*n* = 14) samples included two newly collected individuals from Russia and 12 from the database [21, 22, 24]. Genomic DNA was extracted from tissue samples (liver, spleen, kidney, muscle) preserved in ethanol solution using the QIAamp DNA Mini Kit (Qiagen, Hilden, Germany).

### Mitochondrial DNA sequence: cytochrome *b*

Complete mitochondrial cytochrome *b* sequences (*Cytb*; 1140 base pairs [bp]) were performed using semi-nested polymerase chain reaction (PCR), amplifying two fragments (“first half” and “last half”) in the second PCR [26]. The first PCR was performed using the universal primer pair L14724 and H15915 [27]. The thermocycling parameters for the first PCR were 95 °C for 10 min, followed by 35 cycles at 95 °C for 30 s, 50 °C for 30 s, and 60 °C for 30 s. The first PCR mixtures (20 μl) consisted of 2 μl of 10x TaqGold buffer (Applied Biosystems, Foster City, CA, USA), 2 μl of 25 mM MgCl_2_, 0.8 μl of dNTP, 1 μl of primers (1 pmol of each primer), 13.2 μl of deionized water (DW), and 0.1 μl of AmpliTaq-Gold DNA polymerase (Applied Biosystems). The second PCR was carried out using two primer pairs: R-L14724 and SNH655 [8] for the upper section and U-H15916 and SNL497 [8] for the lower section. The thermocycling parameters were 35 cycles at 95 °C for 30 s, 50 °C for 30 s, and 60 °C for 30 s. The second PCR mixtures (20 μl) for the upper section contained 2 μl of 10x TaqGold buffer (Applied Biosystems), 1.5 μl of 25 mM MgCl_2_, 0.8 μl of dNTP, 1 μl of primers (1 pmol of each primer), 13.7 μl of DW, and 0.1 μl of AmpliTaq-Gold DNA polymerase (Applied Biosystems). The lower section contained 2 μl of 10x TaqGold buffer (Applied Biosystems), 2 μl of 25 mM MgCl_2_, 0.8 μl of dNTP, 1 μl of primers (1 pmol of each primer), 13.2 μl of DW, and 0.1 μl of AmpliTaq-Gold DNA polymerase (Applied Biosystems). The PCR products were sequenced using the PRISM Ready Reaction DyeDeoxy Terminator Cycle Sequencing Kit v. 3.1 (Applied Biosystems) and an ABI3130 automated sequencer (Applied Biosystems). The sequences of both strands were determined using universal primers (M13RP1 and − 21 M13; Applied Biosystems) in *Cytb*. The sequences were aligned using MUSCLE implemented in MEGA7 [28]. The published *Cytb* sequences (1140 bp) from 104 additional individuals of *M. robusta* [21] were used to independently assess the population dynamics of this phylogroup.

### Phylogenetic analyses of mtDNA

Phylogenetic trees were constructed based on maximum likelihood (ML) using MEGA ver. 7.0 software [28] with the substitution models TN93 + G + I. The best-fit model was determined using AIC as implemented in MEGA. The levels of genetic variation and divergence were assessed based on nucleotide diversity (π) using Arlequin ver. 3.5.1 software [29].

To investigate the relationships among haplotypes, we constructed a haplotype network of *Cytb* based on a median-joining method [30] with PopArt [31] using the default settings. We performed mismatch distribution analyses [32] and neutrality tests, Tajima’s *D* [33], and Fu’s *F*s [34], using Arlequin 3.5.1 to detect rapid population expansion.

The significance of neutrality was tested with 1000 replicates of a coalescent simulation. The neutrality tests estimate historical population growth, which generally leads to significantly negative Tajima’s *D* and Fu’s *F*s values. When populations experience a sudden expansion, the expected mismatch distribution of nucleotide differences is smooth and unimodal [32, 35, 36]. We tested the validity of the sudden expansion model using a parametric bootstrap approach with 1000 replicates. The expansion parameter tau (τ) was estimated using Arlequin 3.5.1 for each cluster in which signs of sudden demographic expansion were evident. In this method, the sum of the squared deviations (SSD) between the observed distribution and the expected distribution was compared with the SSD between the simulated distribution and the expected distribution for each replicate. The raggedness index (*r*, [37]) was used as a test statistic for the predicted sudden expansion model. The temporal aspect of rapid expansion was assessed using the formula *t* = τ/2*uk*, where *t* is the time since expansion in generations, *k* is the sequence length, and *u* is the evolutionary rate per generation for the entire sequence [35, 38]. The value of *u* was derived from the formula *u* = μg, where *μ* is the evolutionary rate per site per year and *g* is the generation time in years. The time since expansion in years, *T* (= *tg*), was estimated using the formula *T* = τ/2μk.

## Results

### Molecular phylogeny and geographic boundaries

**Fig. 2:**
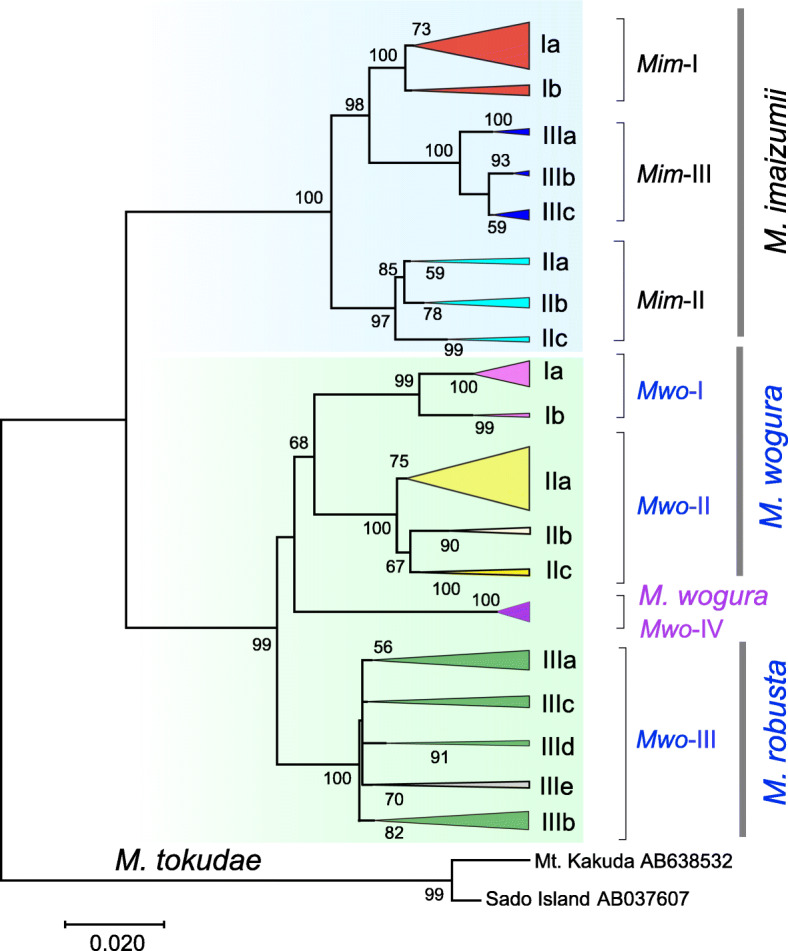
Maximum likelihood tree of cytochrome *b* sequences (1140 bp) from the *Mogera* species. Bootstrap support values > 50% are indicated above the nodes. Clusters in the tree were collapsed by assignment of haplotype groups. The colors of the clusters are the same as those of the locality points in Fig. 1

We constructed an ML tree to illustrate the relationships among the 222 *Cytb* sequences (1140 bp) from two species of Japanese moles, *M. imaizumii* (*n* = 77) and *M. wogura* (*n* = 131; Supplementary Table S1), together with the closely related continental species (*M. robusta*, *n* = 14) using *M. tokudae* as an outgroup (Fig. 2). Inclusion of the additional 104 *M. robusta Cytb* sequences did not alter the tree topology (data not shown). In the phylogenetic analyses, the three phylogroups of *M. imaizumii*, *Mim*-I, -II, and -III, were found to range from the administrative units of Tohoku, Kanto, and Hokuriku-Kansai, respectively (Fig. 1b), as previously reported [5, 14, 22]. The three phylogroups of *M. wogura* had apparent geographic affinity for areas of three administrative units: Kansai/Tokai (*Mwo*-I), Chugoku/Shikoku (*Mwo-*II), and Kyushu (*Mwo*-III), the last of which extended to the western part of the Chugoku District (Hiroshima and Yamaguchi Prefectures; Fig. 1c, d), as was previously reported [5, 22].

Our geographic plots revealed the two boundaries of the mtDNA phylogroups of *M. wogura*: *Mwo*-I vs. *Mwo*-II (border *Mwo*-I/II; Fig. 1c) and *Mwo*-II and *Mwo*-III (border *Mwo*-II/III; Fig. 1c). The *Mwo*-I/II border was located at the northern edge of the Osaka Plain extending north, separating Lake Biwa into western and eastern parts (Fig. 1c). The *Mwo*-I and *Mwo*-II groups corresponded with moles preferring montane and prairie habitats, respectively. In contrast, the *Mwo*-II/III border in Chugoku District was located in Hamada (Localities 75, 76) in Shimane Prefecture and Mihara (Localities 84, 86) in Hiroshima Prefecture and showed no association with any visible physical barriers, such as mountains or rivers.

**Fig. 3:**
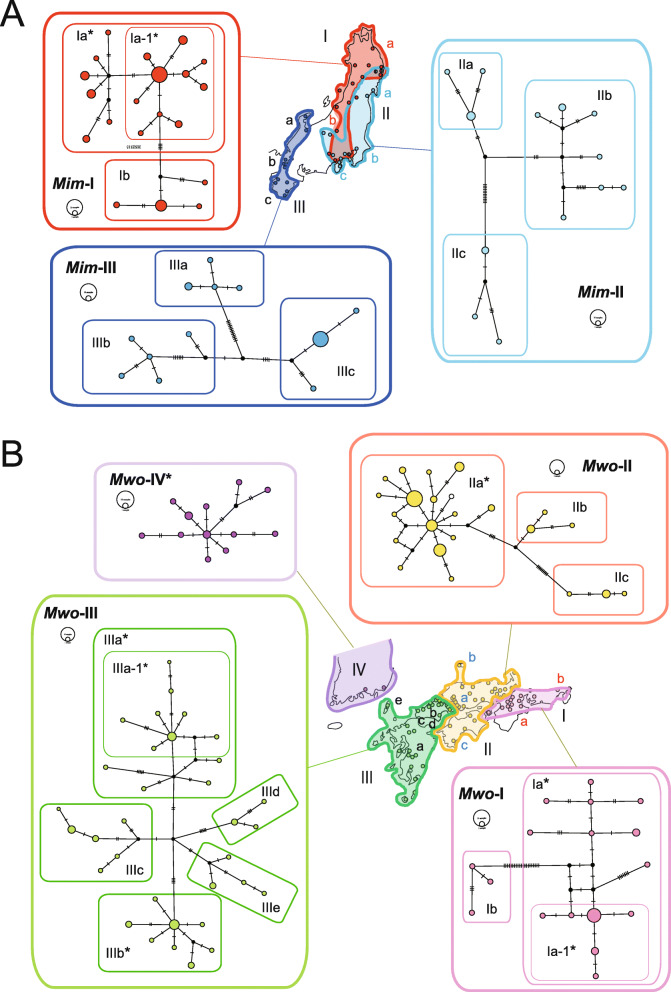
Haplotype networks of cytochrome *b* sequences from *M. imaizumii* (**a**) and *M. wogura* plus *M. robusta* (**b**) using the median-joining method [30] using PopArt. The circle color indicates the intraspecies phylogroup (*Mim*-I, red; *Mim*-II, light blue; *Mim*-III, blue), and the circle size reflects the number of samples. Circles with different colors indicate phylogroups within species of *M. wogura* (*Mwo*-I, pink; *Mwo*-II, yellow; *Mwo*-III, green) and *M. robusta* (*Mwo*-IV, purple). The number of bars on each branch represents the number of substitutions. Haplotype groups are marked with an asterisk when rapid expansion signals were obtained (see Table 1)

### Expansion dynamics of local populations

Median-joining (MJ) network construction revealed a further subdivision in each of the mtDNA phylogroups of the two Japanese mole species. In *M. imaizumii*, *Mim*-I consisted of northern (*Mim*-Ia) and southern (*Mim*-Ib) subgroups, with a border around Niigata (Locality 15 in Fig. 1b). *Mim*-II comprised the subgroups *Mim*-IIa, *Mim*-IIb, and *Mim*-IIc, showing geographic affinity with the Tohoku, Kanto, and Tokai Districts. *Mim*-III showed three subgroups in three geographically separated areas: *Mim*-IIIa (Hokuriku), *Mim*-IIIb (Kansai), and *Mim*-IIIc (southern Kii Peninsula; Figs. 1b, 3a).

The northernmost haplotype group of *M. imaizumii*, *Mim*-Ia (*n* = 36), showed a star-shaped structure containing the subgroup *Mim*-Ia-1 (*n* = 28), which was also a star-shaped structure (Fig. 3a); this suggests that two consecutive events occurred in a short time interval. The mismatch distribution analysis did not reject the sudden expansion model (Table 1), and the neutrality test using Fu’s *F*s yielded significantly negative values in *Mim*-Ia and *Mim*-Ia-1, resulting in τ values of 3.51 and 2.69, respectively (Table 1). *Mim*-II and *Mim*-III, however, were more genetically diverged and showed no star-like pattern.

**Table 1:**
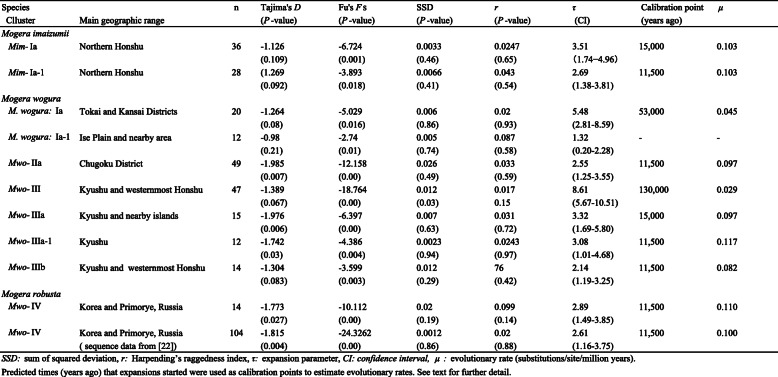
Detection of rapid expansion events and estimation of evolutionary rates of mitochondrial cytochrome *b* gene sequences (1140 bp)

In *M. wogura*, the MJ networks provided evidence of a subdivision of the phylogroups into local haplotype groups (Figs. 1c, 3b). *Mwo*-I included western (*Mwo*-Ia) and eastern (*Mwo*-Ib) geographic subgroups separated by the Fuji River. *Mwo*-II included three subgroups over a broad area of the Chugoku region (*Mwo*-IIa), the Oki Islands plus a confined area of the Chugoku region (*Mwo*-IIb), and Shikoku (*Mwo*-IIc). *Mwo*-III consisted of five subgroups. The largest subgroup (*Mwo*-IIIa) was a cluster on the mainland of Kyushu (*Mwo*-IIIa-1) and haplotypes from two remote islands, Tanegashima (Locality 121 in Fig. 1c) and Yakushima (Locality 122). Haplotypes of the other four subgroups (*Mwo*-IIIb, -IIIc, -IIId, and -IIIe) were mostly located in the westernmost part of Honshu (Fig. 1c). *Mwo*-IIIe consisted of haplotypes from the remote island Tsushima (Locality 109) and five localities in westernmost Honshu (Supplementary Table S1).

The networks showed a star-shaped structure in five sets of haplotype groups in *M. wogura* and *M. robusta*, which were further divided into three classes with respect to the level of variation: small- (τ = 1.32–3.32; *Mwo*-Ia-1, *Mwo*-IIa, *Mwo*-IIIa, *Mwo*-IIIa-1, *Mwo*-IIIb, and *Mwo*-IV), medium- (τ = 5.5; *Mwo*-Ia), and large-value (τ = 8.6; *Mwo*-III) clusters (Fig. 3b, Table 1). The prediction of rapid expansion was supported by a mismatch distribution analysis and one or both neutrality tests (Table 1). A portion of the *Mwo*-Ia haplotypes, which formed a star-shaped cluster designated *Mwo*-Ia-1 and extended around the Ise Plain, had the lowest τ value of 1.32 (CI = 0.20–2.28).

The mtDNA lineage *Mwo*-VI representing the mainland species *M. robusta* (*n* = 14) showed a star-shaped structure in the MJ network (Fig. 3b). The rapid expansion model was supported by a mismatch distribution analysis and both neutrality tests, Tajima’s *D* and Fu’s *F*s, resulting in a τ value of 2.89 (Table 1). We also used a previously reported *Cytb* sequence dataset (*n* = 104, *k* = 1140, [21]) and obtained a τ-value of 2.61, which was similar to the aforementioned results (Table 1).

## Discussion

### Intraspecific spatial structure based on mtDNA variation

In this study, we characterized the genetic features of the mtDNA phylogroups of *M. imaizumii* and *M. wogura* by drawing their geographic ranges. Through extensive sampling efforts, we computed the precise geographic ranges of the mtDNA phylogroups (Fig. 1). The mtDNA boundaries (borders *Mwo*-I/II and *Mwo*-II/III) drawn in this study are generally smooth. It is possible that the mtDNA phylogroups *Mwo*-I, *Mwo*-II, and *Mwo*-III have mutually exclusive relationships that yield these borders.

The *Mwo*-I/II border occurred from Kobe through Osaka to Otsu (the southernmost tip of Lake Biwa) between mountains and plains (Fig. 1c). This finding suggests that the two phylogroups have shifted in their habitat preference. Alternatively, the history of the Osaka Plain, such as the Holocene Jomon transgression [39], may have played a role. At any rate, this provides circumstantial evidence of genetically based local adaptation.

On the other hand, the *Mwo*-II/III border was not associated with any physical barriers, such as mountains or rivers (Fig. 1c). Both phylogroups showed indications of a recent population expansion, as discussed below, and it is possible that the two frontier lines are now facing the border. This result suggests that the two phylogroups have undergone prezygotic isolation and speciation processes to a considerable extent. This idea is supported by their distant position in the phylogenetic tree (Fig. 2). Interestingly, the borders are in similar positions in westernmost Honshu and Kyushu, demarcating the lineages of two geographic groups, including dormice [40], deer [41], harvestman [42, 43], and clouded salamanders [44]. The mechanisms behind the parapatric border positions are not yet understood.

### Population dynamics associated with paleoclimatic fluctuations

The present study of mtDNA variation illuminates the evolutionary history of Japanese moles, especially for the prominent events of sudden population expansion, which are thought to be associated with the drastic environmental fluctuations in the late Quaternary [1, 3, 5, 7, 8, 10, 21, 44] and likely to be major determinants of mtDNA genetic diversity. Moreover, our results provide an opportunity to determine the pattern of the mtDNA evolutionary rate in insectivorous species by providing sequential biogeographic calibration points.

Our mtDNA sequence data show indications of sudden expansion in local populations of *M. wogura* in Honshu and Kyushu. In contrast, whereas the northernmost haplotype group of *M. imaizumii* (*Mim*-I) indicates significant rapid expansion, *Mim*-II and *Mim*-III from more southern regions, i.e., central Honshu and Kansai districts, show no signs of expansion. The trend is similar to the case for Japanese voles, the genus *Myodes*, in which the northern and southern species show contrasting patterns in terms of rapid expansion events in response to late Quaternary environmental fluctuations [3]. *Myodes andersoni*, the northern species, does not show rapid expansion signs, whereas *M. smithii*, the southern species, shows signs of sudden population expansion [3]. This pattern is evident in the presence of the relic populations of northern lineages of both species in western Honshu, southern Honshu, and Shikoku, providing evidence that both northern species extended their territory during colder periods [3, 5]. This finding implies that northern species, i.e., *M. imaizumii* and *My. andersoni*, are cold tolerant.

In this study, we found 10 local haplotype groups having signals of rapid expansion. Contrary to the three groups with distinct τ values of 1.32, 5.48, and 8.61, the remaining seven groups (*Mim*-Ia, *Mim*-Ia-1, *Mwo*-IIa, *Mwo*-IIIa, *Mwo*-IIIa-1, *Mwo*-IIIb, and *Mwo*-IV) showed confined τ values ranging from 2.14 to 3.51 (Table 1). Given the relatively low level of the τ values and the frequent occurrence of expansions over a wide area of the Japanese Archipelago, these values can be assigned to relatively recent historic time points of the post-LGM. These τ values can be divided into two groups, low (τ = 2.14, 2.55, 2.69, 2.89, and 3.08) and high (τ = 3.32 and 3.51), and the latter two haplotype groups (*Mim*-Ia and *Mwo*-IIIa) contain the former groups as subclusters (*Mim*-Ia-1 and *Mwo*-IIIa-1, Fig. 2). Hence, it is conceivable to assign these values to the two prominent post-LGM time points, namely, the end of the last glaciation (ca. 15,000 years ago) and the termination of the Younger Dryas (YD) (ca. 11,500 years ago) ([45, 46]; see Supplementary Figs. S1, S2). The YD cold reversal event was globally synchronous (e.g., [47]), and it was predicted to have affected the population dynamics of terrestrial animals [8, 48]. *Mim*-Ia and *Mwo*-IIIa include haplotypes from remote islands, Awashima Isl. (Locality 14 in Fig. 1b) and the insular groups of Tanegashima and Yakushima (Localities 121 and 122 in Fig. 1c), respectively, which are now separated by the sea (e.g., ~ 120 m; Supplementary Fig. S2). This result therefore suggests that the expansion event of the former involved gene flow through land bridges that are thought to have been retained at the end of the last glaciation period and deconstructed in the YD age [39, 49–52].

The network pattern of the Kyushu group (*Mwo*-III) was star shaped with a τ value of 8.61 (Fig. 3c). This pattern is attributable to a rapid expansion event associated with the end of PGM approximately 130,000 years ago (Table 1; Supplementary Fig. S1 [53]). This idea is supported by the fact that this group contains an insular lineage of Tsushima that is now separated from Kyushu by the deep strait (− 100 m), suggestive of the involvement of gene flow at the time when the sea level was substantially low. The Kyushu group retains multiple lines (*Mwo*-IIIb, IIIc, IIId, IIIe) in Chugoku but only one (*Mwo*-IIIa) in mainland Kyushu, possibly due to the dramatic volcanic activity in Kyushu from the PGM to LGM that presumably reduced genetic diversity. For example, the catastrophic large caldera eruption of Aira, south Kyushu, occurred 29,000–26,000 years ago [54]. It is necessary to verify the possibility of the influence of these eruptions in the future.

*Mwo*-Ia exhibits a star-shaped cluster (Fig. 3) with rapid expansion signals and a τ value of 5.48. The level of the τ value should be an event in the period between the LGM and PGM, suggesting that early MIS 3 is most likely for the third calibration point, accounting for the predicted impact of the glaciation and subsequent warming period [3, 7].

This study provides valuable information for understanding patterns of change in the evolutionary rate of mole mtDNA over the past 150,000 years. The results mentioned above indicate that one can set calibration points at 130,000, 53,000, 15,000, and 11,500 years ago for the termination of the PGM, MIS 4, the last glaciation, and YD, resulting in evolutionary rates (on average) of 0.029, 0.045, 0.10, and 0.10 substitutions/site/myr, respectively (Table 1), which are indicative of time dependency. The evolutionary rate calculated for the relatively old calibration point is roughly consistent with the magnitude of the evolutionary rate of 0.024–0.026 substitutions/site/myr inferred from our previous phylogenetic analysis of major phylogroups within the mole species [5]. The time-dependent evolutionary rates are overall similar between rodents and moles based on the comparison of τ values over time (Supplementary Fig. S1).

The evolutionary rate is likely to be the same at the calibration points 15,000 and 11,500 years ago. This result is congruent with those seen in the cases of voles [3] and wood mice [7, 8]. In addition, a recent study of house mice showed that the evolutionary history of the past 15,000 years, which was reconstructed at an evolutionary rate of 0.10 substitutions/site/myr, is consistent with existing archeological knowledge and is relevant to prehistoric agricultural development in East Asia [55]. Accordingly, the evolutionary rate in such shallow divergence times seems to be constant, and we estimated the expansion starting time of *Mwo*-Ia-1 (Aichi-Kansai, τ = 1.32) with an evolutionary rate of 0.10 substitutions/site/myr of moles, resulting in a time of 5800 years ago (Table 1). The mid-Holocene expansion may represent responses to anthropogenic changes (e.g., development of paddy fields) or natural events (e.g., the Jomon Transgression, [39]). The formation of the plains after transgression (approximately 4000 years ago, [49]) may have initiated the *Mwo*-Ia-1 expansion events.

We examined the population dynamics of the continental lineage *Mwo*-IV recovered from *M. robusta* (also classified as *M. wogura*, [5, 22]) using the current dataset (*n* = 14) and that of the databases (*n* = 104, [21]). The lineage showed a pattern of rapid expansion signals in which the events were estimated to have started approximately 11,500 years ago (Table 1), suggesting that this pattern corresponds to the end of the YD and the subsequent period of rapid warming. This finding indicates that there were synchronized population fluctuations in the Japanese Archipelago and the coastal areas of the continent. This trend has also been reported in Eurasian jays, which consume acorns as a major food resource [56].

## Conclusions

In this study, we addressed the potential driving forces of late Quaternary environmental changes that have shaped the genetic diversity of animals in and around the Japanese Archipelago. We examined mtDNA variations in the phylogroups of three mole species, *M. imaizumii*, *M. wogura*, and *M. robusta*, and provided insight into the phylogenetic status of the mtDNA phylogroups. We hypothesize that many of the phylogroups are in the process of speciation (“*in statu nascendi*”), creating relatively smooth geographic boundaries between them, which should be addressed in future studies using nuclear gene markers. We confirmed that late and middle Quaternary environmental fluctuations were fundamental in shaping the present-day genetic diversity of Japanese mole species. In particular, the end of the last glacial period (ca. 15,000 years ago) and YD (ca. 11,500 years ago) are presumed to have had tremendous impacts on mtDNA sequence variations in these mole species from Japan and the nearest continental region. Our data also show that the marked drop in sea level (~ 120 m) mediated migration events between islands that are currently separated by deep sea areas. The sudden population expansion and connection between islands serve as efficient calibration points to estimate the rates of evolution. The time-dependent evolutionary rates obtained from rodent studies likely explain the mtDNA diversity of the moles, suggesting that the time-dependent evolutionary rate of nucleotide substitutions in *Cytb* can be similar between rodents and moles (Supplementary Fig. S1). This study opens new possibilities in phylogeography and population dynamics studies in moles, rodents, and other mammals. It is plausible to say that the Japanese Archipelago is an ideal site for assessing the time-dependent evolutionary rate of terrestrial taxa [57]. Finally, we would like to emphasize the mole as a useful study system for evaluating the impact of late Quaternary environmental changes on biodiversity and understanding the effects of local environmental changes.

## Supplementary Information

**Additional file 1: Table S1.** Specimens of *Mogera imaizumii*, *Mogera wogura*, and *Mogera robusta* used for molecular analyses in this study.

**Additional file 2: Figure S1. a**. The marine oxygen isotope curve over the last 150,000 years, adapted from Lisiecki and Raymo (2005) [51], with indication of the marine isotope stage (MIS). The four critical periods for small mammal population dynamics in Japan and the nearest continental areas are shown. Abrupt warmings after substantially cold periods are marked with letters (*a*, *b*, *c*, *d*) and boxes of different colors; those immediately after the end of the Younger Dryas (YD; *a*, light blue), the end of the last glacial period (*b*, dark blue), the early MIS 3 (*c,* green), and MIS 5e (*d*, orange) [3, 7, 8]. **b** The detailed climatic fluctuations (source: climate.gov/sites/default/files/default/files/historictemperaturerecord_greenland_large.jpg, download 20 May 15) are shown with the two prominent time periods: YD (*a*) and LGM (*b*). **c**-**f** Plots of the τ values obtained from previous studies of *Apodemus speciosus* (**c**), *A. argentesus* (**d**), *Myodes* voles (**e**) [3, 7, 8], and the present study of *Mogera* moles (**f**). The geographic map shows the approximate distribution of each haplotype group.

**Additional file 3: Figure S2.** A possible link between the population dynamics of Kyushu moles and late Quaternary environmental fluctuations. **a** An MJ network of the *Cytb* sequence dataset of moles from Kyushu and two southern peripheral islands of Tanegashima and Yakushima, showing two star-shaped clusters, termed *Mwo-*IIIa and *Mwo-*IIIa-1, indicative of rapid expansion events that are predicted to have occurred ca. 11,600 (Stage *a*) and ca. 15,000 (Stage *b*) years ago, respectively. **b** A schematic representation of the expanded land mass during the last glacial period approximately 16,000 years ago, when the sea level was ~ 120 m lower than at present [50]. The broad line shows the estimated coastline at the last glacial maximum [49]. **c** Global sea level change for the last 35,000 years, covering the last glacial maximum (LGM) and Younger Dryas (YD) [51, 52]. Arrows indicate the presumed time points of Stages *a* and *b*, when the two rapid expansion events of the mitochondrial gene haplogroups of *Mwo-*IIIa-1 and *Mwo-*IIIa are thought to have initiated in the mainland of Kyushu and the region that also included the two islands, respectively.

## Data Availability

Newly obtained DNA sequences were deposited in the DDBJ/EMBL/GenBank database with accession numbers LC554619-LC554753. DNA alignments have been deposited in Dryad: 10.5061/dryad.cjsxksn51.
