# Supplementary material for: Influence of Quaternary environmental changes on mole populations inferred from mitochondrial sequences and evolutionary rate estimation
Source: Zoological Lett. 2021 Feb 15;7:2. doi: 10.1186/s40851-021-00169-9 (PMC7885446; doi:10.1186/s40851-021-00169-9)
Supplement: Supplementary file 1 — Additional file 1: Table S1. Specimens of Mogera imaizumii, Mogera wogura, and Mogera robusta used for molecular analyses in this study. [file 40851_2021_169_MOESM1_ESM.pdf]

Supplementary Table S1. Specimens of *Mogera imaizumii, Mogera wogura*, and *Mogera robusta* used for molecular analyses in this study

Species	Locality No.		Collection locality	Specimen code	*Cytb* haplogroup
Mogera imaizumii	1	Aomori	Hachinohe	KT2793/HS499;AB037611*	*Mim*-Ia
	2	Akita	Akita	MH14257/HS5786	*Mim*-Ia-1
	3		Yuzawa	MH14258/HS5787	*Mim*-Ia-1
				MH14259/HS5788	*Mim*-Ia-1
				MH14263/HS5792	*Mim*-Ia-1
	4	Iwate	Morioka	HEG319;AB245936**	*Mim*-Ia
				HEG320;AB245958**	*Mim*-Ia-1
				HEG321;AB245937**	*Mim*-Ia-1
				HEG322;AB245938**	*Mim*-Ia-1
				HEG323;AB245939**	*Mim*-Ia-1
				HEG350;AB245940**	*Mim*-Ia
	5		Miyako	HS1777;AB245960**	*Mim*-Ia-1
	6		Otsuchi	HEG198;AB245948 **	*Mim*-Ia-1
				HEG170;AB245941**	*Mim*-Ia-1
				HEG172/HS1498;AB245942**	*Mim*-IIa
				HEG352;AB245943**	*Mim*-Ia
				HEG353;AB245944**	*Mim*-Ia
				HEG354;AB245945**	*Mim*-Ia
				HEG180/HS1514;AB245946**	*Mim*-Ia
				HEG344;AB245947**	*Mim*-Ia-1
				HEG347;AB245951**	*Mim*-Ia-1
				HEG197/HS1508;AB245948**	*Mim*-Ia-1
				HEG200;AB245949**	*Mim*-Ia-1
				HEG348;AB245950**	*Mim*-Ia-1
				HEG325;AB245962**	*Mim*-Ia-1
				HEG326;AB245955**	*Mim*-Ia-1
				HEG327;AB245954**	*Mim*-Ia
	7		Tono	HEG346;AB245952**	*Mim*-IIa
				HS2315;AB245961**	*Mim*-Ia-1
	8		Kamaishi	HEG181/HS1515;AB245956**	*Mim*-Ia-1
				HEG328;AB245953**	*Mim*-Ia-1
	9	Yamagata	Tsuruoka	MH14264/HS5793	*Mim*-Ia
				MH14265/HS5794	*Mim*-Ia
	10		Kamio	MH14266/HS5795	*Mim*-Ia-1
				MH14267/HS5796	*Mim*-Ia-1
				MH14268/HS5797	*Mim*-Ia-1
				MH14269/HS5798	*Mim*-Ia-1
	11		Ogunimachi	MH14270/HS5799	*Mim*-Ib
	12	Miyagi	Ishinomaki	HEG175;AB245957**	*Mim*-IIa
				HEG343;AB245963**	*Mim*-IIa
	13		Sendai	HS582;AB037613*	*Mim*-IIa
	14	Niigata	Awashima I.	HS1521;AB638494***	*Mim*-Ia
	15		Igarashi	HS470; AB037610*	*Mim*-Ib
				KT2795/HS364;AB270529**	*Mim*-Ib
	16	Gunma	Minakami	HS3057	*Mim*-Ib
				KT3331/HS3077	*Mim*-Ib
	17	Nagano	Chikuma	KT4014/HS4572	*Mim*-IIb
	18		Karuizawa	HS1778;AB245959**	*Mim*-IIb
	19	Ibaraki	Tsukubamirai	KT4469/HS5630	*Mim*-IIb
				KT4470/HS5631	*Mim*-IIb
				KT4471/HS5632	*Mim*-IIb
	20	Yamanashi	Ichikawamisato	HS4491	*Mim*-Ib
	21		Aokigaharajukai	KT3468/HS3187	*Mim*-IIc
	22	Kanagawa	Yokohama	HS472	*Mim*-Ib
				KT2697/HS365;AB037616*	*Mim*-IIb
	23		Atsugi	HS3896;AB638496***	*Mim*-IIb
	24		Hakone	HS3092	*Mim*-IIc
	25	Shizuoka	Ito	HS1022*	*Mim*-IIc
	26		Mishima	KT3469/HS3184	*Mim*-IIc
	27		Shizuoka	SIK:0775;HG737872****	*Mim*-Ib
	29	Toyama	Toyama	HS5456	*Mim*-IIIa
				HS5457	*Mim*-IIIa
				HS5458	*Mim*-IIIa
				HS5462	*Mim*-IIIa
	30	Ishikawa	Noto	HS3366;AB638495***	*Mim*-IIIa
	37	Shiga	Takashima	HS3367;AB638497***	*Mim*-IIIb
				HS3368; LC554207***	*Mim*-IIIb
	45	Kyoto	Mt. Hiei	M13285/HS4736	*Mim*-IIIc
	46		Iwakurahasemachi	HS463; AB037619*	*Mim*-IIIb
	47		Nagaokakyo	HS1843	*Mim*-IIIb
	48	Osaka	Kawachinagano	MH14237/HS5757	*Mim*-IIIc
			Kawachinagano	MH14253/HS5783	*Mim*-IIIc
	49	Nara	Mt. Wasamata	MH9967/HS4620	*Mim*-IIIc
	50	Wakayama	Kumanogawa-cho	KT2707/HS368;LC554208***	*Mim*-IIIc
	51		Kozagawa-cho	MH14218/HS5758	*Mim*-IIIc
				MH14219/HS5759	*Mim*-IIIc
	52		Wakayama	KT2807/HS462	*Mim*-IIIc

(to be continued)

Supplementary Table S1. Specimens of *Mogera imaizumii* and *Mogera wogura* used for molecular analyses in this study (continued)

Species	Locality No.	Collection Locality		Specimen Code	*Cytb* haplogroup
Mogera wogura	24	Kanagawa	Hakone	KT3491/HS3096;AB638498***	*Mwo*-Ib
	26	Shizuoka	Mishima	KT3201/HS1023;AB037623*	*Mwo*-Ib
	28		Fujinomiya	TUA1519/HS3897;AB638499***	*Mwo*-Ib
	31	Aichi	Okazaki	HS4429;AB638500***	***Mwo*-Ia**
	32		Kasugai	HS581 ; AB037625*,***	***Mwo*-Ia**
	33	Fukui	Nadasyomura	NH11483/HS4776	*Mwo*-IIa
	34	Shiga	Takashima	MH11604/HS4884	*Mwo*-IIa
	35		Otsu	MH11720/HS4925	***Mwo*-Ia-1**
				MH11721/HS4926	***Mwo*-Ia-1**
				MH11722/HS4927	***Mwo*-Ia-1**
	36		Kusatsu	MH13056/HS5625	***Mwo*-Ia-1**
				MH43057/HS5626	***Mwo*-Ia-1**
	38	Mie	Fujiwara-cho, Ogaito	MH11723/HS4932	***Mwo*-Ia-1**
	39		Komono-cho, Otowa	MH11726/HS4935	***Mwo*-Ia-1**
	40		Iga, Onogi	MH11717/HS4929	***Mwo*-Ia-1**
				MH11712/HS4930	***Mwo*-Ia-1**
				MH11713/HS4931	***Mwo*-Ia**
	41	Nara	Nara	MH11530/HS4898	***Mwo*-Ia**
	42		Ojimachi	MH14245/HS5762	***Mwo*-Ia-1**
				MH14246/HS5764	***Mwo*-Ia-1**
	43		Koryo-cho	MH11463/HS4764	***Mwo*-Ia-2**
	44		Mt. Abe	MH11517/HS4894	***Mwo*-Ia-1**
				MH11518/HS4895	***Mwo*-Ia-1**
	53	Osaka	Kawachinagano	KT2767/HS314;AB638503***	***Mwo*-Ia**
				MH7937/HS4623	***Mwo*-Ia**
	54	Kyoto	Kameoka	MH11474/HS4766	*Mwo*-IIa
				MH11476/HS4767	*Mwo*-IIa
	55		Ashiu	HS683;AB638501***	*Mwo*-IIa
				HS810;AB638502***	*Mwo*-IIa
	56		Kyotanba-cho, Wachi	MH11482/HS4774	*Mwo*-IIa
	57		Fukuchiyama	MH11477/HS4768	*Mwo*-IIa
	58	Hyogo	Toyooka	MH11298/HS4690	*Mwo*-IIa
	59		Tanba	MH11478/HS4769	*Mwo*-IIa
				MH11479/HS4770	*Mwo*-IIa
	60		Sasayama	MH11480/HS4771	*Mwo*-IIa
				MH11481/HS4772	*Mwo*-IIa
	61		Takarazuka	MH11271/HS4642	***Mwo*-Ia**
	62		Arimaonsen	MH11269/HS4641	*Mwo*-IIa
	63		Kobe	HS4664	*Mwo*-IIa
	64		Aioi	HS4582	*Mwo*-IIa
	65		Awaji Is.	MH11279/HS4657	***Mwo*-Ia**
	66	Tottori	Misasa-cho	MH14180/HS5629	*Mwo*-IIa
	67		Yonago	HA56115/HS4432;AB638505***	*Mwo*-IIa
	68	Okayama	Maniwa	MH14176/HS5627	*Mwo*-IIa
				MH414177/HS5628	*Mwo*-IIa
	69		Akaiwa	HS4585	*Mwo*-IIa
				HS4586	*Mwo*-IIa
	70	Simane	Oki Is.	HA56101/HS4433;AB638504***	*Mwo*-IIb
	71		Gotsu, Matsukawa	MH13052/HS5153	*Mwo*-IIa
				MH13053/HS5154	*Mwo*-IIa
	72		Gotsu, Ninomiya	MH13054/HS5155	*Mwo*-IIb
				MH13055/HS5156	*Mwo*-IIb
	73		Gotsu, Nagaya	MH13407/HS5342	*Mwo*-IIa
				MH13408/HS5343	*Mwo*-IIa
	74		Hamada, Kanagi	MH13605/HS5362	*Mwo*-IIa
				MH13606/HS5363	*Mwo*-IIb
	75		Hamada, Kochi	MH13603/HS5360	*Mwo*-IIa
				MH13604/HS5361	*Mwo*-IIa
	76		Hamada, Sufu	MH14024/HS5597	*Mwo*-IIIa
	77		Masuda, Uchida	MH13681/HS5423	*Mwo*-IIIb
	78		Masuda, Umetsuki	MH13689/HS5421	*Mwo*-IIIb
				MH13690/HS5422	*Mwo*-IIIe
	79	Hiroshima	Hiwa-cho	HA55944/HS4430;AB638506***	*Mwo*-IIa
	80		Miyoshi, Kimita	MH14017/HS5479	*Mwo*-IIa
				MH14018/HS5480	*Mwo*-IIa
	81		Miyoshi, Mirasaka	MH14023/HS5485	*Mwo*-IIa
	82		Miyoshi, Kisa	MH14019/HS5481	*Mwo*-IIa
				MH14020/HS5482	*Mwo*-IIa
				MH14021/HS5483	*Mwo*-IIa
				MH14022/HS5484	*Mwo*-IIa
	83		Sera-cho, Uzuto	MH14043/HS5604	*Mwo*-IIa
				MH14044/HS5605	*Mwo*-IIa
	84		Fuchu-cho	MH13042/HS5143	*Mwo*-IIa
				MH13043/HS5144	*Mwo*-IIa
				MH13044/HS5145	*Mwo*-IIa
	85		Fukuyama, Kannabe	HS5425	*Mwo*-IIa
	86		Mihara, Daiwa	MH13347/HS5323	*Mwo*-IIIb

(to be continued)

Supplementary Table S1. Specimens of *Mogera imaizumii* and *Mogera wogura* used for molecular analyses in this study (continued)

Species	Locality No.	Collection Locality		Specimen Code	*Cytb* haplogroup
	87	Hiroshima	Mihara, Kui	MH14045/HS5606	*Mwo*-IIa
				MH14046/HS5607	*Mwo*-IIIb
	88		Higashihiroshima,Toyosaka	HS5424	*Mwo*-IIa
	89		Miyoshi, Miwa	MH43614/HS5369	*Mwo*-IIIe
	90		Akitakata, Koda	MH13613/HS5368	*Mwo*-IIIe
	91		Akitakata, Yoshida	MH13612/HS5367	*Mwo*-IIId
	92		Akitakata, Midori	MH13045/HS5146	*Mwo*-IIa
				MH13046/HS5147	*Mwo*-IIa
	93		Akitakata, Yachiyo	MH13047/HS5148	*Mwo*-IIId
	94		Kitahiroshima-cho, Oasa	MH13409/HS5344	*Mwo*-IIa
				MH13410/HS5345	*Mwo*-IIb
	95		Kitahiroshima-cho, Totani	MH13608/HS5365	*Mwo*-IIIc
				MH13609/HS5366	*Mwo*-IIIc
	96		Akiota, Tahara	MH13607/HS5364	*Mwo*-IIIb
	97		Akiota, Togochi	HA55940/HS4449;AB638507***	*Mwo*-IIIb
				HA55943/HS4450;AB638508***	*Mwo*-IIIe
	98		Hiroshima, Shiraki	MH13048/HS5149	*Mwo*-IIId
				MH13049/HS5150	*Mwo*-IIId
	99		Higashihiroshima	MH13346/HS5322	*Mwo*-IIa
			Higashihiroshima	HS5324	*Mwo*-IIIe
	100	Yamaguchi	Abu-cho, Mizugasako	MH12401/HS5157	*Mwo*-IIIb
	101		Waki-cho	MH11524/HS4899	*Mwo*-IIIb
				MH11525/HS4900	*Mwo*-IIIb
	102		Nagato	MH11084/HS4606	*Mwo*-IIIc
				MH11528/HS4896	*Mwo*-IIIb
				MH11529/HS4897	*Mwo*-IIIc
	103		Shimonoseki	HS4448;AB638509***	*Mwo*-IIIb
				MH11526/HS4901	*Mwo*-IIIb
				MH11527/HS4902	*Mwo*-IIIc
				MH14047/HS5608	*Mwo*-IIIb
	104	Kagawa	Mannou	MH11782/HS4951	*Mwo*-IIc
	105	Tokushima	Mima	MH5921/HS4605	*Mwo*-IIc
	106	Kochi	Kagamiogachi	MH11778/HS4947	*Mwo*-IIc
	107		Shimantonakamura	MH11780/HS4949	*Mwo*-IIc
	108	Ehime	Iyo	HS5500	*Mwo*-IIc
	109	Nagasaki	Tsushima Is.	KT2711/HS366;AB638510***	*Mwo*-IIIe
				KT3473/HS2820;AB638511***	*Mwo*-IIIe
				HA55911/HS4434;AB638512***	*Mwo*-IIIe
	110	Fukuoka	Fukuoka	HS5501	*Mwo*-IIIb
	111		Ukiha	HA55915/HS4437;AB638513***	*Mwo*-IIIa
				HA55916/HS4438;AB638514***	*Mwo*-IIIa
	112		Kurume	HA55919/HS4439;AB638515***	*Mwo*-IIIa
	113	Kumamoto	Yatsushiro	HA55922/HS4441;AB638519***	*Mwo*-IIIa
	114		Amakusa	YK05/HS4428;AB638518***	*Mwo*-IIIa
	115		Ashikita	HA55926/HS4442;AB638517***	*Mwo*-IIIa
	116		Kuma	HS414;AB638516***	*Mwo*-IIIa
	117		Hitoyoshi	HA55932/HS4446;AB638520***	*Mwo*-IIIa
				HS4447;AB638521****	*Mwo*-IIIa
	118	Miyazaki	Kiyotake	KT3276/HS3066;AB638522***	*Mwo*-IIIa
	119	Kagoshima	Makizono	KT2699/HS369;LC554209***	*Mwo*-IIIa
	120		Uchinoura	HS415;LC554210***	*Mwo*-IIIa
	121		Tanegashima Is	HA55930/HS4445;AB638523***	*Mwo*-IIIa
	122		Yakushima Is.	HA55926/HS4443;AB638524***	*Mwo*-IIIa
				HA55928/HS4444;AB638525***	*Mwo*-IIIa
M. robusta	123	Russia	Krasnoarmeysky	AK884;AB638530***	*Mwo*-IV
	124		Ussuriisk	AK820/HS2004;AB638528***	*Mwo*-IV
				AK821/HS2005;AB638529***	*Mwo*-IV
	125		Shkotovo	HS2009	*Mwo*-IV
	126		Kedrovaya Pad	AK001/HS890*	*Mwo*-IV
				AK002/HS1170; AB037646*	*Mwo*-IV
				AK609/HS2001	
	127		Hasan	AK707/HS1392;AB638527***	*Mwo*-IV
	128	China	Liaoning	LN111102;HG737874****	*Mwo*-IV
				LN111103;HG737875****	*Mwo*-IV
				LN111104;HG737873****	*Mwo*-IV
	129	Korea	Seoraksan	HS930; AB037641*,***	*Mwo*-IV
	130		Pusan	KT2755/HS373;AB638526***	*Mwo*-IV
	131		Namphae	HS929;AB037640*,***	*Mwo*-IV

*Tsuchiya et al. (2000), **Iwasa et al. (2006), ***Kirihara et al. (2013), ****He et al. (2014)

Cytb haplotypes of Ia are shown in bold letters and those of Ia-1 in underlined.
