# Supplementary figures and images for: Influence of Quaternary environmental changes on mole populations inferred from mitochondrial sequences and evolutionary rate estimation
Source: Zoological Lett. 2021 Feb 15;7:2. doi: 10.1186/s40851-021-00169-9 (PMC7885446; doi:10.1186/s40851-021-00169-9)

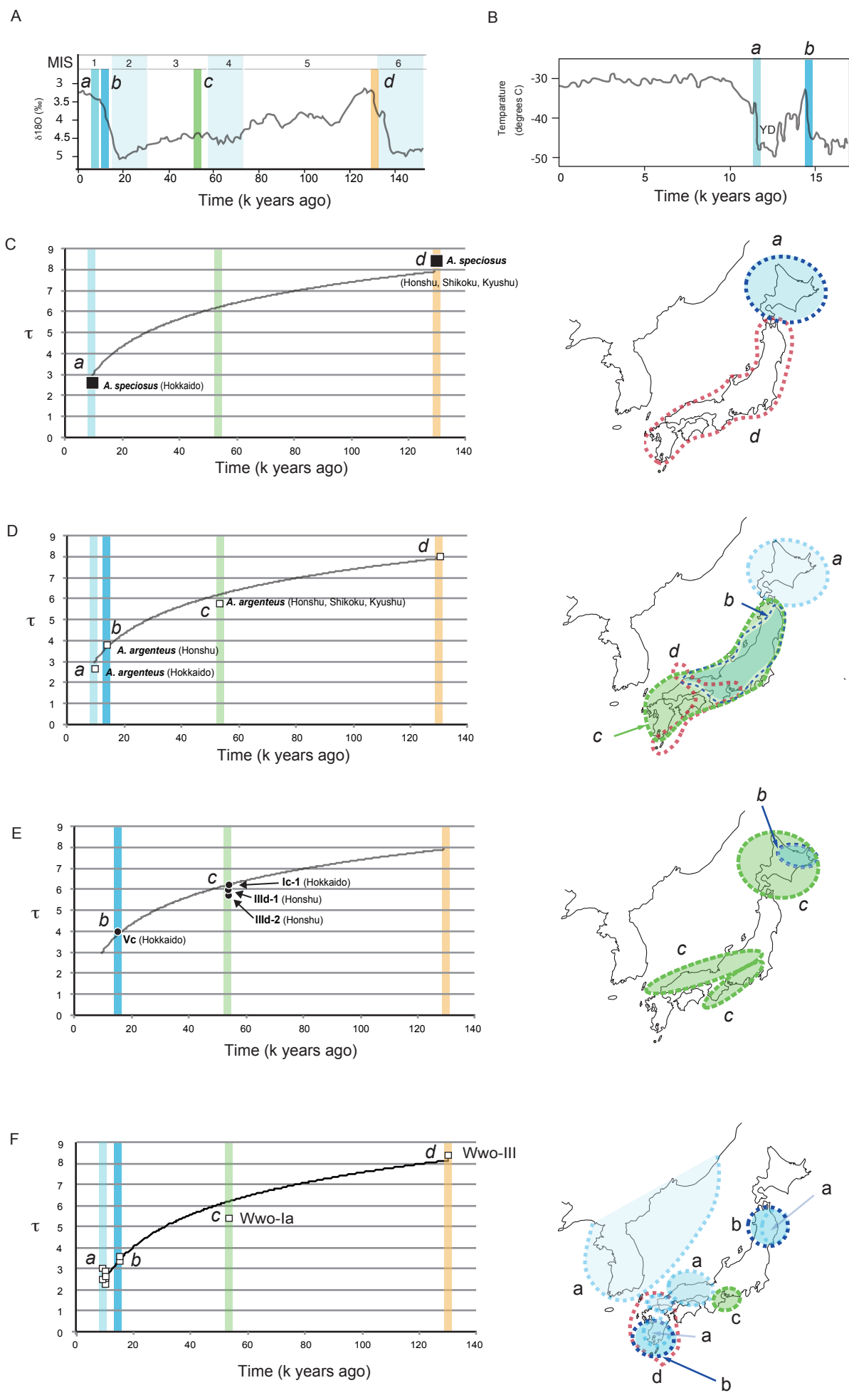

Supplementary Fig. S1

Supplement: Supplementary file 2 — Additional file 2: Figure S1. a. The marine oxygen isotope curve over the last 150,000 years, adapted from Lisiecki and Raymo (2005) [51], with indication of the marine isotope stage (MIS). The four critical periods for small mammal population dynamics in Japan and the nearest continental areas are shown. Abrupt warmings after substantially cold periods are marked with letters (a, b, c, d) and boxes of different colors; those immediately after the end of the Younger Dryas (YD; a, light blue), the end of the last glacial period (b, dark blue), the early MIS 3 (c, green), and MIS 5e (d, orange) [3, 7, 8]. b The detailed climatic fluctuations (source: climate.gov/sites/default/files/default/files/historictemperaturerecord_greenland_large.jpg, download 20 May 15) are shown with the two prominent time periods: YD (a) and LGM (b). c-f Plots of the τ values obtained from previous studies of Apodemus speciosus (c), A. argentesus (d), Myodes voles (e) [3, 7, 8], and the present study of Mogera moles (f). The geographic map shows the approximate distribution of each haplotype group. [file 40851_2021_169_MOESM2_ESM.pdf]

A

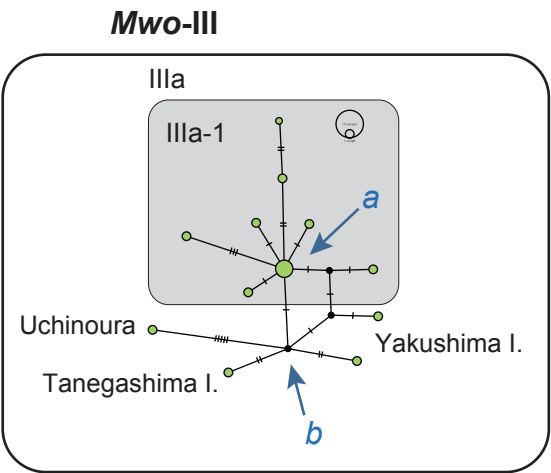

B

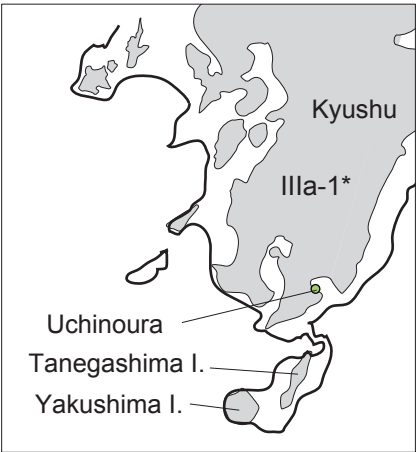

C

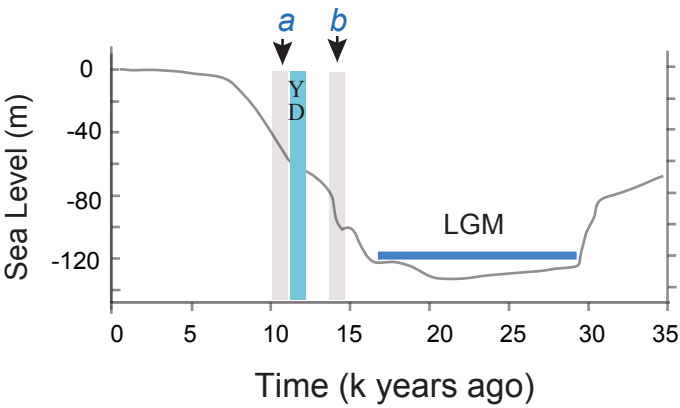

Supplement: Supplementary file 3 — Additional file 3: Figure S2. A possible link between the population dynamics of Kyushu moles and late Quaternary environmental fluctuations. a An MJ network of the Cytb sequence dataset of moles from Kyushu and two southern peripheral islands of Tanegashima and Yakushima, showing two star-shaped clusters, termed Mwo-IIIa and Mwo-IIIa-1, indicative of rapid expansion events that are predicted to have occurred ca. 11,600 (Stage a) and ca. 15,000 (Stage b) years ago, respectively. b A schematic representation of the expanded land mass during the last glacial period approximately 16,000 years ago, when the sea level was ~ 120 m lower than at present [50]. The broad line shows the estimated coastline at the last glacial maximum [49]. c Global sea level change for the last 35,000 years, covering the last glacial maximum (LGM) and Younger Dryas (YD) [51, 52]. Arrows indicate the presumed time points of Stages a and b, when the two rapid expansion events of the mitochondrial gene haplogroups of Mwo-IIIa-1 and Mwo-IIIa are thought to have initiated in the mainland of Kyushu and the region that also included the two islands, respectively. [file 40851_2021_169_MOESM3_ESM.pdf]
